# The Effects of Acupuncture on Glutamatergic Neurotransmission in Depression, Anxiety, Schizophrenia, and Alzheimer's Disease: A Review of the Literature

**DOI:** 10.3389/fpsyt.2019.00014

**Published:** 2019-02-12

**Authors:** Cheng-Hao Tu, Iona MacDonald, Yi-Hung Chen

**Affiliations:** ^1^Graduate Institute of Acupuncture Science, China Medical University, Taichung, Taiwan; ^2^Department of Photonics and Communication Engineering, Asia University, Taichung, Taiwan; ^3^Chinese Medicine Research Center, China Medical University, Taichung, Taiwan

**Keywords:** acupuncture, glutamate, neuropsychiatric disorders, Alzheimer's disease, depression, anxiety, schizophrenia

## Abstract

Neuropsychiatric disorders, including depression, anxiety, schizophrenia, and Alzheimer's disease (AD), are diseases that are directly or indirectly associated with cerebral dysfunction and contribute significantly to disability in adult populations worldwide. Important limitations surround the currently available pharmacologic agents for neuropsychiatric disorders and, moreover, many patients fail to respond to these therapies. Acupuncture might be a complementary therapy for neuropsychiatry disorders. In this review, we investigate the current evidence for the treatment efficacy of acupuncture in depression, anxiety, schizophrenia, and AD. Secondly, we review recent advances in understanding of the dysregulated glutamate system underlying the pathophysiology of these disorders. Finally, we discuss the ways in which acupuncture treatment can potentially modulate glutamate receptors and excitatory amino acid transporters. We conclude that the treatment effects of acupuncture may be underpinned by its intervention in the dysregulated glutamate system. Further preclinical and clinical studies are needed to clarify the possible mechanisms of acupuncture in these neuropsychiatric disorders and to establish protocols for treatment guidelines.

## Introduction

Neuropsychiatry focuses on illness relating to altered cognition, mood, or behavior caused by cerebral dysfunction with neuronal pathological changes (e.g., dysregulated neurotransmitter systems or tissue damage), or abnormal physiological conditions (e.g., hyper/hypoglycemia or hypoxia). These changes in health conditions have profound effects upon individuals and society (1). Neuropsychiatric disorders contribute to over 10% of disability worldwide, exceeding the morbidity rates associated with cardiovascular disease or cancer (2). In developed-market economies, 25% of all disability has been attributed to neuropsychiatric disorders (2). In 2016, ~18% of adults aged ≥18 years in the United States of America had any mental illness in the past year and ~4% had a serious mental illness in that period (3, 4). A serious mental illness is defined as a diagnosable mental, behavioral, or emotional disorder (e.g., major depressive disorder [MDD], schizophrenia) causing serious functional impairment and substantially interfering with or limiting one or more major life activities, such as maintaining interpersonal relationships, activities of daily living, self-care, employment, and recreation (3, 4). Similarly, a systematic review of epidemiological data from 16 European countries estimated in 2005 that 27% of the adult population (18–65 years of age) had experienced at least one mental disorder (e.g., substance use, psychosis, depression, anxiety, or eating disorder) in the past 12 months (5). Despite the high burden of psychiatric illness, only a subset of these people receive the mental health services that they need. For instance, according to data from the World Health Organization (WHO) European Region, 3 out of 4 people with MDD are inadequately treated (6). The majority of psychiatric disorders are mild or moderate, but if left untreated, the evidence suggests that they can develop into more serious illness (2).

Important limitations surround the currently available treatments for neuropsychiatric disorders. For instance, first-line pharmacotherapy for MDD typically consists of a selective serotonin reuptake inhibitor, a serotonin and norepinephrine reuptake inhibitor, or the norepinephrine-dopamine reuptake inhibitor bupropion, alone or in combination with psychotherapy. These therapies are associated with low remission rates and high dropout rates (7). As for the treatment of schizophrenia, the typical and atypical antipsychotics are mostly effective for the positive symptoms (hallucinations, delusions); cognitive and negative symptoms (deficits in working memory and attention, negative affect, and anhedonia) are largely unresponsive to current pharmacologic therapies. Moreover, serious side effects limit the use of some otherwise effective medications (8).

## Acupuncture in Neuropsychiatry Treatment

Acupuncture has long been used in Chinese medicine to treat numerous neuropsychiatric conditions, from acute delirium to post-stroke spasticity. The two forms of acupuncture manipulation that are used clinically are manual acupuncture (MA) and electroacupuncture (EA) (9). Traditional acupuncturists commonly use MA, whereby they insert the acupuncture needle into the acupoint to a certain depth and rotate it by hand. In EA, the needles are connected to an electrical stimulator that delivers a stimulating current to the acupoints. Another method that is also described as EA is the positioning of a surface electrode on the skin at the acupoint, without insertion of acupuncture needles. For this review, the evidence on the use of acupuncture in neuropsychiatry treatment is limited to investigations using MA and EA, with acupuncture needle insertion.

Numerous clinical reports from various sources, including the non-Western scientific literature, attest to the efficacy of acupuncture in depression (10–13), anxiety disorders (14, 15), schizophrenia (16–19), and Alzheimer's disease (AD) (20–23). Although much of this evidence is widely acknowledged to be of varying quality, many reports attest to the efficacy and safety of acupuncture treatment (24–27). In experienced hands, acupuncture is a safe therapy with a low risk of adverse events. Serious and potentially life-threatening acupuncture-related complications, including transmission of infections, pneumothorax, cardiovascular lesions, and hemorrhage or hematomas in the central nervous system (CNS), are very rarely reported (28). In a large study from Germany that included 2.2 million acupuncture sessions in 229,230 patients, the overall incidence of acupuncture-related adverse events was 8.6%, among which 2.2% of the patients required medical treatment (29). The vast majority of adverse events were due to minor bleeding/hematomas (6.1%), pain (1.7%), or vegetative symptoms such as vertigo, or nausea (0.7%). A review of three Chinese trials involving nearly 2,000 treatments identified instances of subcutaneous hematoma, bleeding and needle site pain, and reported that elderly people seem to be at greater risk of such adverse events (30).

### Acupuncture for the Treatment of Depression

The effectiveness of acupuncture in depression has been extensively investigated with various sets of acupuncture points and treatment parameters (e.g., duration, frequency, and number of treatment sessions). A recent Cochrane systematic review that included 64 studies (7,104 participants) examined the effectiveness of acupuncture for the treatment of depression (10). The evidence indicated that acupuncture treatment may moderately or slightly lower the severity of depression compared with treatment as usual and control acupuncture (invasive, non-invasive sham controls), respectively, although the quality of the evidence was judged as being mostly low or very low. Other reviews have also described evidence in support of MA and EA as generally beneficial, safe and well-tolerated as monotherapy in MDD and post-stroke depression, but the evidence is insufficient to support the use of acupuncture in combination with antidepressants (31, 32). Two Chinese studies examined the effectiveness of acupuncture and moxibustion (a treatment involving burning of the dried Chinese herb mugwort or *Artemesia vulgaris* to apply heat onto or very close to an acupoint) in relieving psychological distress in 163 patients with depression and sought to determine whether gender-related differences exist in response to acupuncture and moxibustion (12, 13). The authors reported that acupuncture and moxibustion can significantly improve distress at even as late as 3 months after the completion of treatment, and that the level of efficacy is higher among females than males. Hence, acupuncture treatment in depression may improve depressive symptoms of depression and endure for some months.

Moreover, sleep status may improve in patients with depression after acupuncture treatment. A recent meta-analysis that included 18 randomized clinical trials (RCTs) involved 1,678 adults given acupuncture for depression-related insomnia and found significant improvements in sleep quality with acupuncture compared with Western medicine (11). When acupuncture was given as an adjunctive therapy with Western medicine, both depression and insomnia were improved (11). However, in another RCT involving 150 patients with residual insomnia associated with MDD, traditional acupuncture needling produced only mild treatment effects that were similar to those of minimal acupuncture and placebo acupuncture (33). There were no significant group-by-time interactions during the 5-week post-treatment period. Thus, the psychological effect of acupuncture might play an important role in the treatment of acupuncture in depression-related insomnia.

### Acupuncture for the Treatment of Anxiety

The effectiveness of acupuncture in anxiety has been widely investigated, using various sets of acupuncture points and treatment parameters. A narrative review published by the British Acupuncture Council reported that regular acupuncture and EA treatments improved anxiety symptoms (34). However, significant differences between the protocols used in regular acupuncture and EA made it hard to rule out a general beneficial or possible placebo effect. A more recent systematic literature review that included 32 English-language clinical and preclinical studies published between 2000 and 2010 also reported significant, positive results with acupuncture treatment for anxiety (14). Although the quality of these studies was variable, the authors suggested that patients who are resistant to conventional interventions (e.g., cognitive behavioral therapy) may prefer acupuncture treatment. Thus, acupuncture treatment may have positive effects on the symptoms of anxiety.

Acupuncture may also be of benefit in anxiety-related insomnia. A Canadian study reported that acupuncture significantly improved sleep quality in patients with anxiety and insomnia (15). At the end of acupuncture treatment, urine 6-sulfatoxymelatonin (a metabolite of melatonin) levels were normalized and several polysomnographic measures, as well as self-reported fatigue, sleepiness, anxiety, and level of depression, were significantly improved. Combined with the evidence of treatment efficacy in depression-related insomnia, acupuncture may have broader utility in neuropsychiatric disorders with impaired quality of sleep.

Interestingly, acupuncture treatment may also reduce anxiety levels in medical conditions other than neuropsychiatry. An early study reported that auricular acupuncture reduced state anxiety in patients waiting for surgery by a significantly greater extent than either body or sham acupuncture (35). Another study has reported that both body and auricular acupuncture effectively reduce preoperative anxiety; self-rated anxiety scores were significantly reduced from baseline in both groups (36). Possible underlying mechanisms remain unclear. One plausible explanation is that acupuncture regulates the autonomic nervous system. For instance, acupuncture appears to modulate heart rate variability, a non-invasive indicator of changes in autonomic state. In patients with mild depression or anxiety, verum acupuncture but not sham acupuncture was associated with significant reductions from baseline in mean resting heart rate at 5 and 15 min after needle application, with a trend toward an increase in high frequency (HF; 0.15–0.4 Hz) and a decrease in low frequency (LF; 0.04–0.15 Hz) spectral power (37). These results suggest that verum acupuncture modulates autonomic activity in response to alterations of internal and external environments, and thus reduces overall anxiety in patients with depression or anxiety.

However, it should be recognized that the interpretation of results from clinical trials investigating acupuncture interventions in depression and anxiety disorders is complicated by different interventions, different comparators used against acupuncture interventions, and the small sample sizes in many trials (38). These shortcomings prevent any accurate assessment of acupuncture for these conditions or a true comparison of the relative effectiveness of different treatment regimens. Moreover, the data are difficult to interpret from those studies where needling at specific and non-specific points have yielded similar outcomes (38).

The evidence consulted for depression and anxiety is summarized in Table 1.

**Table 1:** Overview of characteristics of included studies evaluating acupuncture for depression and anxiety.

**Smith et al. (10)**	
Participants	7,104 adult men and women with depression.
Interventions	This Cochrane Review included 64 RCTs comparing acupuncture vs. control acupuncture (invasive, non-invasive sham controls), no treatment/wait-list, medication, psychological therapy, or standard care. Modes of treatment included acupuncture, EA and laser acupuncture.
Outcomes	Acupuncture may moderately reduce the severity of depression when compared with treatment as usual/no treatment. Use of acupuncture may reduce the severity of depression in comparison with control acupuncture. The very low quality of evidence limits the interpretation of the effects of acupuncture vs. medication and psychological therapy. Risks of adverse events with acupuncture are also unclear, as most trials did not report adverse events.
	Review paper
**Fan et al. (13)**	
Participants	163 patients with depression.
Interventions	Participants were randomized either to acupuncture plus moxibustion using a method that soothes the liver and regulates the mind (Group A; *n* = 54), to an acupoint shallow puncturing group with a shorter duration of moxibustion (Group B; *n* = 56), or to a non-acupoint shallow puncturing group (Group C; *n* = 53). All participants received twice-weekly treatment for 12 weeks. Group A received conventional acupuncture at the 4 gate points [Hegu [LI4]; Taichong [LR3]; Baihui [GV20]; Yintang [GV29]], with moxibustion applied with a moxa cone directly to the 4 flower points (Geshu [BL17]; Danshu [BL19]). Group A also received intradermal needling at Xinshu (BL15) and Ganshu (BL18). For Group B, the same acupoints were used as those for Group A, but with a shallower needling depth and a shorter duration of moxibustion. Group C received acupuncture at acupoints 10 mm lateral to the acupoints used in Group A, with the same manipulation method as in Group B.
Outcomes	At 1 and 3 months after treatment, several Symptom Checklist 90 (SCL-90) scores were improved from baseline by a significantly greater extent in Group A compared with Group C (all p < 0.05). SCL-90 scores for depression, anxiety and hostility were improved by a significantly greater extent at 1 and 3 months in Group A compared with Group B (all p < 0.05).
**Fan et al. (12)**	
Participants	163 patients with depression.
Interventions	Acupuncture and moxibustion soothing liver and regulating mind treatment (Group A); acupoint shallow puncturing (Group B); and non-acupoint shallow puncturing (Group C). Acupoints used in Group A were bilateral Hegu (LI4), bilateral Taichong (LR3), Baihui (GV20), and Yingtang (GV29). Needles were inserted vertically to a depth of 10–12 mm for LI4 and LR3; the needles were inserted at an angle of 30° to a depth of 4–5 mm for GV20 and GV29. After recording the *de qi* needling sensation (e.g., numbness, soreness, distention, heaviness during acupuncture stimulation), acupuncture used the *dao qi* technique at the acupoints, involving lifting, thrusting, and rotation of the needle. Needles remained in place for 30 min. Moxibustion involved the four flower points (bilateral BL17 and BL19). A round moxa cone (1 cm diameter, 1 cm high) was placed and ignited on the selected points, which were smeared evenly with Wanhua oil. Five cones were applied to each point. Bilateral BL15 Xinshu and BL18 Ganshu were selected for intradermal needling. An intradermal needle was completely inserted at each point and retained for 2 days. All treatments were given twice weekly for 12 weeks, with intervals lasting more than 48 h between two treatments. For Group B, the same acupoints were used as those in Group A, with a 2- to 3-mm needling depth. No lifting, thrusting, or rotating was performed, and the needle sensation was not required. Moxibustion was done for a shorter duration and only 1–2 mm of the intradermal needle body was inserted into the points. Group C received acupuncture at points 10 mm lateral to LI4 and LR3 and 10 mm to the left side of GV20 and GV29 during the acupuncture procedure, 10 mm lateral to BL17 and BL19 during the moxibustion procedure, and 10 mm lateral to BL15 and BL18 during the intradermal needling procedure, with the same manipulation method as that in Group B.
Outcomes	At 1 and 3 months after treatment, SCL-90 and HAMD scores differed by sex between Group A and Group B; women were more sensitive to the efficacy of the soothing liver and regulating mind treatment compared with other methods.
**Dong et al. (11)**	
Participants	1,678 adults (aged 18–75 years) with depression-related insomnia.
Interventions	11 RCTs compared acupuncture with Western medicine; 5 RCTs compared medication alone or in combination with acupuncture; 2 RCTs compared acupuncture with sham or placebo acupuncture control.
Outcomes	A pooled analysis of 10 RCTs that reported PSQI scores demonstrated significant improvements with acupuncture over Western medicine. When acupuncture was combined with Western medicine, sleep quality, and depression were both improved by a greater extent compared with Western medicine alone. Improvements from baseline in HAMD scores did not differ significantly between acupuncture treatment and Western medicine.
	Review paper
**Chung et al. (33)**	
Participants	150 outpatients with residual insomnia associated with MDD.
Interventions	Nine × 30-min sessions of acupuncture, given 3 times a week for 3 consecutive weeks. Participants were asked to continue the same type and dosage of antidepressants throughout the study period. They were randomized to “Traditional acupuncture” based on TCM principles of acupuncture treatment for insomnia, minimal acupuncture, or placebo acupuncture. The traditional acupuncture group was needled at bilateral Ear Shenmen, Sishencong (EX-HN1), Anmian (EX), Neiguan (PC6), Shenmen (HT7), Sanyinjiao (SP6), and unilateral Yintang (EX-HN3) and Baihui (GV20), using the TCM style of acupuncture. Acupoints on the head, hands and legs were treated with 0.25 × 25-mm needles; ear acupoints on ears were treated with 0.20 × 25-mm needles; insertion depths varied between 2 and 25 mm, depending on the points selected. *De qi* was achieved if possible. All needles were connected to an electric stimulator that delivered a constant current (0.4 ms, square-wave, brief-pulse stimulus of 4-Hz frequency). The needles were left for 30 min and then removed.
	The minimal acupuncture group was needled at points that have no therapeutic effects according to TCM theory and superficially to avoid *de qi*. Points on the limbs included bilateral “forearm,” 1 inch lateral to the middle point between Shaohai (HE3) and Shenmen (HE7); “upper arm,” 1 inch lateral to Tianfu (LU 3); and “lower leg,” 0.5 inch dorsal to Xuanzhong (GB39). Points on the head included bilateral “head,” the middle point between Shuaigu (GB8) and Touwei (ST8); “forehead”, the middle point between Touwei (ST8) and Yangbai (GB14); “neck”, the middle point between Tianyou (TB16) and Tianrong (SI17); and “ear,” a point on the helix, inferior to the apex. Other treatment conditions and electrostimulation were the same as in the acupuncture group. The placebo acupuncture group received Streitberger placebo needles placed at sites 1 inch beside the acupuncture points used in the acupuncture group, with the aim of avoiding an acupressure effect. The needles were connected to an electric stimulator but with zero frequency and amplitude.
Outcomes	Traditional acupuncture needling produced only mild hypnotic effects that were similar to those of minimal acupuncture and placebo acupuncture. A high proportion of patients in each treatment group remained clinically significantly affected by insomnia after treatment.
**British Acupuncture Council (34)**	
Participants	Patients with anxiety disorders or depression.
Interventions	Verum (regular) acupuncture compared with sham acupuncture and EA.
Outcomes	Sham-controlled studies indicated that anxiety improved with both regular acupuncture and EA treatments. Significant differences between the protocols used in regular acupuncture and EA precluded any general beneficial or possible placebo effect. Moreover, although the findings from most controlled studies indicated a general anxiety-reducing effect of acupuncture, these were regarded by the reviewers as inconclusive because of study design problems, including the absence of standardized symptom rating scales in most studies, limited follow-up, and poorly defined differences between protocols used in different studies.
	Review book
**Errington-Evans (14)**	
Participants	Healthy volunteers, patients with anxiety disorders, and animal models of anxiety, from 32 English-language articles published between 2000 and 2010.
Interventions	TCM and non-TCM acupoints were used in patients, with a lack of detail provided by the studies as to point selection and treatment methodology. Animal studies assessed treatment outcomes in rodents subjected to chronic mild stress (controls) vs. no stress (a “natural” group), acupuncture vs. sham acupuncture.
Outcomes	The poor quality of the methodology reporting prevents any treatment recommendation.
	Review paper
**Spence et al. (15)**	
Participants	18 adult volunteers reporting having symptoms of insomnia for ≥2 continuous years immediately prior to the study and with scores >50 (anxiety range) on the Zung Anxiety Self Rating Scale. The study participants did not satisfy DSM-IV criteria for any particular anxiety disorder.
Interventions	Acupuncture therapy was given for 5 weeks (2 sessions/week, 10 sessions in total). Each acupuncture session lasted ~1 h. Two consecutive overnight polysomnographic studies were performed at baseline (before treatment) and at the end of the acupuncture treatment. Mood and cognitive efficiency was tested by the Toronto Alexithymia Scale, the Stanford Sleepiness Scale (SSS), and a 7-item Fatigue Scale. Anxiety was assessed by the State-Trait Anxiety Inventory and depressive symptoms by the Center for Epidemiological Studies Depression Scale (CES-D). On the following morning, immediately after waking, each subject completed a standard post-sleep questionnaire, the SSS, and the Fatigue Scale. Approximately 20 min after awakening, subjects assessed their level of fatigue and sleepiness on the Fatigue Severity Scale, the Epworth Sleepiness Scale, the Toronto Western Hospital Fatigue Questionnaire, the Fatigue Scale, and the FaST Adjective Checklist. They were also tested for accuracy and time to complete a complex verbal reasoning task. During both test phases, urine samples were tested for changes in endogenous levels of melatonin over 24 h.
Outcomes	At 5 weeks, acupuncture treatment was associated with significant reductions in state and trait anxiety scores, significant improvements in polysomnographic measures of sleep onset latency, arousal index, total sleep time, and sleep efficiency. Nocturnal endogenous melatonin secretion was significantly increased.
Acupoints	No mention of acupoints.
**Wang and Kain (35)**	
Participants	55 patients with preoperative anxiety.
Interventions	Participants were randomized to 1 of 3 groups: bilateral auricular acupuncture protocol at the Shenmen point (*n* = 22); bilateral auricular acupuncture at a “relaxation” point (a protocol believed to be effective against anxiety) (*n* = 15); or acupuncture at a sham acupuncture point (*n* = 18). Press-acupuncture needles were inserted at the respective auricular areas and remained in place for 48 h.
Outcomes	The Relaxation group was significantly less anxious at 30 min and at 24 and 48 h compared with the other 2 groups.
**Wu et al. (36)**	
Participants	35 healthy adult volunteers with preoperative anxiety.
Interventions	Participants received either auricular acupuncture at the Shenmen point, to a depth of about 0.2 cm (*n* = 18) or body acupuncture at 4 acupoints, to a depth of nearly 0.5 cm (*n* = 17). The acupuncture needles were 25–40 mm long and 0.25–0.30 mm in diameter. All subjects received twice-weekly treatments over 4 weeks (8 sessions in all). Each acupuncture session lasted approximately 30 min.
Outcomes	Scores on the Zung Self-Rating Anxiety Scale were significantly reduced from baseline in both groups.
**Agelink et al. (37)**	
Participants	36 patients with mild depression or anxiety disorder.
Interventions	Nine acupuncture sessions involving classical acupuncture points (He7, Pe6, Du20, Bl62, Ex6), or sham acupuncture (needles were applied epidermally at non-acupuncture points).
Outcomes	Compared with sham acupuncture, verum acupuncture was associated with a significant decrease in the mean resting heart rate and a significant decrease in the mean low LF:HF ratio.

### Acupuncture for the Treatment of Schizophrenia

Compared with depression and anxiety, relatively few studies have addressed the efficacy of acupuncture in schizophrenia. A meta-analysis of 13 RCTs including 954 patients, all from China, provides positive evidence for the effectiveness of acupuncture (with or without EA or moxibustion) in treating the symptoms of schizophrenia (19). Some of the RCTs reported that acupuncture plus drug therapy significantly improved auditory hallucinations, positive symptoms and response rates compared with antipsychotics alone or in combination with sham EA. In a recent systematic review of data from 26 studies (1,181 participants) reporting limited evidence for the use of adjunctive acupuncture therapy in the treatment of positive, negative and cognitive symptoms, the authors point out the importance of differences between quantitative and qualitative changes (16). The limited evidence for treatment efficacy may be partly due to the fact that positive symptoms still exist, but patients are suffering less. Other evidence suggests that individualized acupuncture is beneficial for patients with schizophrenia as an adjunctive treatment with routine care (18). After completing individualized acupuncture sessions, patients reported improvements in symptoms of schizophrenia, side effects of medication, energy, motivation, sleep, addictions and other associated physical problems. A case study has reported that positive and negative symptoms can be improved for up to 3 months after add-on acupuncture treatment (17). A more recent case study describes how add-on acupuncture treatment improved general psychopathology and negative symptoms but not positive symptoms (39). Thus, individualized add-on acupuncture treatment may help to alleviate symptoms of schizophrenia.

Interestingly, the review by van den Noort et al. also describes beneficial effects with add-on acupuncture in accompanying sleep disorders (16). Several of the studies in that review reported improvements in subjective and objective sleep measurements. Acupuncture treatment appears to have similar effects to but is safer than zopiclone, a prescription medication for sleep disorder. Thus, acupuncture treatment may improve sleep dysregulation in schizophrenia.

As with the clinical evidence for acupuncture in depression and anxiety disorders, the clinical evidence is limited for the effectiveness of acupuncture as a treatment for schizophrenia. The meta-analysis performed by Lee and colleagues found that all of the included studies were limited by low methodological quality, the low overall number, and the small sample sizes (19). Moreover, as all of the studies were conducted in China, international trials are needed to investigate whether the effects can be replicated in other ethnicities.

The evidence consulted for schizophrenia is summarized in Table 2.

**Table 2:** Overview of characteristics of included studies evaluating acupuncture for schizophrenia.

**Lee et al. (19)**	
Participants	Chinese patients with schizophrenia.
Interventions	EA plus pharmacological therapy vs. sham EA plus pharmacological therapy. Acupuncture treatment vs. antipsychotics. Acupuncture plus antipsychotics vs. antipsychotics alone. Laser acupuncture vs. sham laser acupuncture.
Outcomes	In one study, EA plus drug therapy significantly improved auditory hallucinations and positive symptoms compared with sham EA plus drug therapy. In 4 studies, acupuncture significantly improved response rates compared with antipsychotics. In 7 studies, acupuncture plus antipsychotics significantly improved response rates compared with antipsychotics alone. One study reported that laser acupuncture ameliorated hallucination. Another study showed significant effects of laser acupuncture on response rates, BPRS and clinical global index scores compared with sham laser. The total number of RCTs included in the analysis, the total sample size, and methodological quality were too low to draw firm conclusions.
	Review paper
**van den Noort et al. (16)**	
Participants	1,181 patients with schizophrenia in regular care receiving acupuncture as add-on therapy.
Interventions	Only studies that used mechanical acupuncture in the treatment of patients with schizophrenia were included in this systematic review.
Outcomes	Most of the 26 identified studies had limited evidence for the use of acupuncture as add-on therapy in the treatment of positive, negative, and cognitive symptoms. Beneficial effects were reported for subjective and objective sleep measurements.
	Review paper
**Ronan et al. (18)**	
Participants	11 patients with schizophrenia.
Interventions	10-week course of individualized acupuncture treatment as an adjunct to routine care in schizophrenia.
Outcomes	All participants reported improvements in symptoms of schizophrenia, side effects of medication, energy, motivation, sleep, addictions, and other associated physical problems.
**Bosch et al. (17)**	
Participants	63-year-old woman with chronic schizophrenia.
Interventions	12 weekly acupuncture treatments, in addition to medication for chronic schizophrenia. Clinical diagnostic interviews and psychological testing (on sleep quality, depression, positive, and negative symptoms) were conducted before, immediately after and 3 months after the acupuncture treatment. The woman received individualized acupuncture treatment once weekly for 12 weeks, bilaterally for each point. The needles used were 0.25 × 25 or 0.20 × 15 mm stainless steel (depending on the place of needling) single-use needles and were placed according to TCM principles. After obtaining *de qi*, the needles were not stimulated thereafter, but left in place for 1 h.
Outcomes	The woman reported improved daily functioning and became less disturbed by her hallucinations. Her pain levels were also markedly reduced. Sleep improved immediately with acupuncture treatment. At 3 months after acupuncture treatment, positive and negative symptoms had decreased and depression scores had improved.
**Bosch et al. (39)**	
Participants	42-year-old man with chronic schizophrenia and co-morbid sleep disorders.
Interventions	In addition to his ongoing Western pharmacotherapy for schizophrenia, the man was treated for 12 weeks with acupuncture treatment in the clinic. The following acupuncture points were selected (with the absolute and the relative frequencies of use in parentheses) for use during the 12 weekly acupuncture treatments: Lidui (ST45) (12 = 100%); Sishencong (EX-HN 1) (11 = 92%); Zhaohai (KI6) (10 = 83%); Shencang (KI25) (7 = 58%); Baihui (DU20) (5 = 42%); Guanyuan (CV4) (4 = 33%); Zhiyin (BL67) (3 = 25%); Yutang (CV18) (3 = 25%); Wenliu (LI7) (2 = 17%); Taixi (KI3) (1 = 8%); Lieque (LU7) (1 = 8%); Taiyang (EX-HN 5) (1 = 8%); Tianshu (ST25) (1 = 8%); Yingu (KI10) (1 = 8%); Xiyan (eye of the knee) (1 = 8%); Shaofu (HT8) (1 = 8%).
Outcomes	The TCM diagnosis of a Liver Fire pattern before acupuncture was not as marked after the 12^th^ acupuncture session. The patient had a small improvement in negative symptoms and in his general psychopathology, accompanied by a small reduction in the number of depressive symptoms. He experienced a marked improvement in sleep disorders following acupuncture, and actiwatch data revealed he was moving less during sleep.

### Acupuncture for the Treatment of Alzheimer's Disease

Another disorder that commonly exhibits neuropsychiatric symptoms is dementia. Neuropsychiatric symptoms are amongst the earliest signs and symptoms of neurocognitive disorders and incipient cognitive decline, and can be challenging to treat (40). Evidence shows that acupuncture can increase a patient's verbal and motor skills and improve mood and cognitive function. In an early Chinese trial, 38 patients with senile dementia were treated with acupuncture and acupoint-injection with aceglutamide (23). After treatment, symptoms had improved in 16 patients. In a small pilot study, 1 month of acupuncture treatment improved cognitive function in 8 patients with mild-to-moderate AD (22). Mini-Mental State Examination subscores assessing verbal orientation and motor coordination, as well as overall scores, were significantly improved from baseline. In a US study that included 11 patients with dementia (10 with AD and 1 with vascular dementia), depression and anxiety scores improved significantly after acupuncture treatment (21). These early pilot studies indicated that acupuncture treatment may be of benefit for neuropsychiatric symptoms in dementia, especially in AD.

More recently, a larger clinical trial involving 87 patients with mild-to-moderate AD reported that acupuncture may significantly improve cognitive function (20). When used as monotherapy, acupuncture treatment was associated with significantly greater decreases from baseline in Alzheimer's disease Assessment Scale-Cognitive (ADAS-cog) scores compared with donepezil. The improvement in cognitive function was observed for up to 12 weeks after the end of acupuncture treatment. Moreover, no patients discontinued treatment because of adverse events in the acupuncture group, but four patients did so in the donepezil group. In addition, a small-scale functional magnetic resonance imaging study explored the relationships between *de qi* sensations (a special needling sensation evoked by acupuncture) induced by different needling depths of acupuncture and their differential effects on the reorganizations of whole-brain networks in 12 patients with mild cognitive impairment (41). The results show that as compared with superficial needling, acupuncture with deep needling induces stronger, wider-ranging *de qi* sensations and enhances nodal centrality, primarily in the abnormal regions of the brain implicated in mild cognitive impairment. Hence, acupuncture treatment in mild-to-moderate AD appears to be not only beneficial but also safe, with the capacity to improve dysfunctional neural mechanisms involved in mild cognitive impairment.

The evidence consulted for AD is summarized in Table 3.

**Table 3:** Overview of characteristics of included studies evaluating acupuncture for Alzheimer's disease.

**Chen (23)**	
Participants	38 outpatients with senile dementia (SDAT, *n* = 17; MID, *n* = 21). A control group consisted of 20 elderly people (average age 69 years) with normal intelligence and mental functioning.
Interventions	Treatment with acupuncture and acupoint-injection with aceglutamide (1 ml, usually given after acupuncture). The acupuncture needles were retained in the selected acupoints for 20 min and were given every other day, for 3 courses of 15 sessions each. The acupoints chosen for needling were mainly in the Governor Vessel, such as Baihui (GV20), Naohu (GV17), Shuigou (GV26), etc., and aceglutamide was injected into tonic points, including Dazhui (GV14), Ganshu (BL18), Shenshu (BL23), Zusanli (ST36), etc. Acupoint injection generally followed acupuncture treatment.
Outcomes	After 45 sessions, improvement (improvement from baseline in HDS or FAQ scores and a general improvement in symptoms) was observed in 7 SDAT cases and 9 MID cases; treatment was excellent (HDS score increased by ≥2 grades or approached normal and FAQ score also approached normal) in 2 MID cases and effective (increase of 1 grade in HDS score and a considerable increase in FAQ score) in 7 MID cases.
**Kao et al. (22)**	
Participants	8 patients with mild-to-moderate AD.
Interventions	8 acupoints were selected according to the China National Standards on Acupoints (GB 12346-90): the Sishencong (Estra 6, 4 points on the scalp), Shenmen (HT7 on both wrists) and Taixi (KI3 on both feet). Needles were inserted to a depth of 0.5 inches at an angle into the Sishengcong, >0.5 inches directly into the Shenmen and 0.8 inches directly into the Taixi. Needling at each acupoint lasted for 30 min in total, comprising the needle testing and its re-insertion after every 10 min of needle therapy. Acupuncture was given in a 7-day treatment cycle with a 3-day break in-between for a total of 30 days.
Outcomes	Acupuncture was associated with significant improvements from baseline in cognition, as assessed by scores on the Mini Mental State Examination (MMSE) measuring verbal orientation (p < 0.01), motor coordination (p < 0.05) and overall score (p < 0.05). Acupuncture also produced a significant overall clinical improvement from baseline (p < 0.05) on the TCM Symptoms Checklist for AD.
**Lombardo et al. (21)**	
Participants	11 patients (10 with AD and 1 with vascular dementia).
Interventions	Patients received acupuncture twice weekly for 3 months; each patient had a minimum of 22 treatments. The initial 10 main acupoints selected were GB9, GV16, GV20, GV23, GV24, PC6, HT7, SP6, Sishencong, and Yintang. Secondary points selected included ST36, LI4, GB20, GV17, SP4, KI3, SI3, BL62, BL23, GV26, and the cervical and thoracic Huato Jiaji points.
Outcomes	Acupuncture was associated with statistically significant improvements in depression and anxiety scores. Some patients also experienced improvements in cognitive function.
**Jia et al. (20)**	
Participants	87 patients with mild-to-moderate AD.
Interventions	Acupuncture 3 times weekly for 12 weeks or once-daily donepezil 5 mg for 4 weeks then 10 mg/day for a further 8 weeks. No other treatments for AD were allowed during the study. Sterile, disposable needles (diameter, 0.25 mm; length, 40 mm) were used at the following acupoints: RN17 (danzhong), RN12 (zhongwan), RN6 (qihai), ST36 (zusanli), SJ5 (waiguan), and SP10 (xuehai). The following acupoints could be selected as auxiliary acupoints according to a patient's symptoms and tongue manifestation: LR3 (taichong), GB39 (xuanzhong), ST40 (fenglong), BL17 (geshu), ST44 (neiting), ST25 (tianshu), and RN4 (guan yu an). Except for RN17, RN12, RN6, and RN4, all other acupoints were bilateral. Acupuncture prescriptions were individualized to each patient, and different points were used based on the discretion of the acupuncturist. The acupuncture achieved *de qi*. To evoke needle sensation, the needles were inserted obliquely and upward 15 mm into RN17, 15–25 mm perpendicularly into RN12, RN6, and ST36, then rotated at small-amplitude and high frequency with a reinforcing method for 30 s. The needles were inserted perpendicularly 15–25 mm into SJ5, then rotated with normal reinforcement and normal reduction method for 30 s. For SP10, the needle was inserted obliquely 15–25 mm into the acupoint, then rotated with large-amplitude and low-frequency reducing method for 30 s. The needles remained in place for 30 min.
Outcomes	At 28 weeks, ADAS-cog scores were decreased from baseline by a significantly greater amount in the acupuncture group compared with the donepezil group. At weeks 10 and 28, mean CIBIC-Plus values were significantly lower in the acupuncture group vs. the donepezil group. No patients discontinued acupuncture treatment because of adverse events, whereas 4 donepezil recipients did so.
**Bai et al. (41)**	
Participants	12 patients with mild cognitive impairment and 12 age-match normal healthy controls.
Interventions	Each study group received 2 functional runs. They initially underwent a resting state scan for 6 min without any stimulation. Acupuncture was then performed at acupoint KI3 on the right leg (Taixi, located on the medial border of the foot posterior to the medial malleolus, in the depression between the tip of the medial malleolus and the Achilles tendon). The needle was inserted vertically to a depth of 1–2 cm with deep needling (DA), but of 1–2 mm in superficial needling (SA). Each acupuncture paradigm incorporated needle manipulation for 2 min, preceded by 1 min of rest and followed by 6 min of rest (no acupuncture manipulation). The presentation sequence of these 3 runs was randomized throughout the study population. Each participant performed only 1 run daily.
Outcomes	Compared with controls, patients exhibited losses of small-world attributes indicated by longer characteristic path lengths and larger clustering coefficients. Acupuncture with deep needling induced stronger and wider-ranging *de qi* sensations both in intensity and prevalence. Deep needling exhibited a modulatory effect to compensate the losses of small-world attributes in the patients; superficial needling had no such effect. Deep needling also enhanced nodal centrality, primarily in the abnormal regions of the brain of patients, including the hippocampus, postcentral cortex, and anterior cingulate cortex.

## Glutamate and Glycine in CNS Disorders

In the past decade, increasing evidence implicates the important role of glutamate in the pathophysiology of many CNS disorders (42, 43). L-Glutamate, the most common excitatory neurotransmitter in the CNS, is involved in synaptic plasticity and cognition (42, 44, 45). Glutamate receptors, synaptic receptors located primarily on the membranes of neuronal cells, are responsible for the glutamate-mediated postsynaptic excitation of neural cells and are important for neural communication, memory formation, learning, and regulation. Neural glutamate signaling is accommodated by two receptor families: ionotropic glutamate receptors (iGluRs; e.g., the N-methyl-D-aspartate [NMDA] receptor and the α-amino-3-hydroxy-5-methyl-4-isoxazolepropionic acid [AMPA] receptor) and metabotropic glutamate receptors (mGluRs) (46). The iGluRs produce excitatory glutamate-evoked currents, whereas the mGluRs are G protein-coupled receptors that control cellular processes through the G protein signaling cascades. Evidence implicates iGluRs, and/or mGluRs, as potential drug targets in neuropsychiatric disorders, including depression, anxiety, schizophrenia, and AD (46, 47).

One of the iGluRs is the NMDA receptor, which is activated by glutamate as well as glycine as a co-agonist. Its activation allows cations to flow through the cell membrane. The NMDA receptor is very important for controlling synaptic plasticity and memory function (48). Recent studies indicate that the dysregulation of the NMDA receptor may play an important role in schizophrenia (49, 50) and depression (51). Conversely, altered AMPA receptor functioning is a feature of many CNS disorders, including amyotrophic lateral sclerosis (ALS), ischemia, traumatic brain injury, epilepsies, and AD (47). The AMPA receptor is responsible for mediating most of the fast synaptic transmission in the CNS (47). Modulation of AMPA receptor numbers explains much of the plasticity of excitatory transmission in the brain, whereby increasing or decreasing AMPA receptors alters synaptic strength, which may be linked to neuropsychiatric disorders such as schizophrenia and depression (52, 53).

The release of glutamate in the synaptic cleft at a particular concentration is maintained by either glutamine synthetase or excitatory amino acid proteins, i.e., excitatory amino acid transporters (EAATs), which re-uptake excessive glutamate from the synapse. A high density of EAATs near the synapse ensures quick removal or transportation of any unbound glutamate. Of the five different membrane-bound transporters, EAAT2 performs more than 90% of the clearance of extracellular glutamate into crude synaptosomes to prevent neuronal excitotoxicity and hyperexcitability. When this process is impaired, it can allow large amounts of glutamate to spill out from the synapse, which may be a pathophysiological mechanism in CNS disorders (54) including schizophrenia, cognitive deficits, dementia and AD, and other disorders. Evidence suggests that EAAT2 activation is a promising therapeutic approach in many neuropsychiatric disease models (45).

Throughout the CNS, glycine acts as a co-agonist with L-glutamate at NMDA receptors. Glycine fluxes are regulated by two specific glycine transporters: GlyT1 and GlyT2. Whereas, GlyT2 is expressed in the spinal cord, brainstem and cerebellum, GlyT1 is expressed in these regions as well as in the forebrain areas such as the cortex, hippocampus, septum, and thalamus (55). GlyT2 is expressed by glycinergic nerve endings in rat spinal cord, while GlyT1 appears to be preferentially expressed by glial cells. Preclinical investigations suggest that GlyT2 is predominantly responsible for glycine uptake at glycinergic synapses, and that GlyT1 is involved in monitoring glycine concentration surrounding NMDA receptor-expressing synapses. In rats, GlyT1 inhibition potentiates NMDA receptor activity and affects NMDA receptor-dependent long-term potentiation (55). It is possible to modulate the function of the NMDA receptor by varying the availability of the glycine co-agonist. This has potential in the treatment of schizophrenia: the NMDA hypofunction hypothesis of schizophrenia postulates that increasing glutamatergic transmission via the NMDA receptors and inhibiting GlyT1 on glial cells enhances NMDA receptor neurotransmission by slowing the process of removal of glycine from the synapse and thus elevates synaptic glycine levels (55, 56).

### Glutamate, Depression, and Anxiety

The monoamine hypothesis of depression contends that the underlying pathophysiology is due to depleted levels of serotonin, norepinephrine, and/or dopamine in the CNS (57). However, no direct evidence supports a primary dysfunction of a specific monoamine system in patients with MDDs. Moreover, not only do many patients fail to respond to monoamine antidepressants, but residual symptoms, relapses and recurrences are common even with adequate dosing of these medications (58) and it can take up to several days or weeks for core depressive symptoms to begin to lift after monoamine antidepressants have elevated synaptic monoamine levels (57–59). MDD pathogenesis appears to involve something else beyond the monoamine system.

Glutamatergic modulation shows potential in antidepressant treatment. A single subanesthetic intravenous dose of the NMDA receptor antagonist ketamine acts rapidly in treatment-resistant depression within hours of administration, with effects that are typically sustained for 7–14 days (60). Moreover, the ketamine metabolite (2R,6R)-hydroxynorketamine [(2R,6R)-HNK] appears to have the antidepressant effects of ketamine and lacks the psychiatric, psychotomimetic, cardiovascular, neurological, and other side effects associated with acute dosing of ketamine in patients with depression (52). Unlike ketamine, (2R,6R)-HNK is not a NMDA receptor antagonist but is associated with the modulation of the AMPA receptor. Thus, both NMDA and AMPA receptors may be involved in the pathophysiological changes of depression and potentially represent new targets for the development of rapid-acting antidepressants.

The glutamatergic system also plays a major role in the pathogenesis of anxiety (61–63). Long-term administration of various antidepressant agents including selective serotonin reuptake inhibitors (SSRIs), serotonin and norepinephrine reuptake inhibitors (SNRIs), tricyclic antidepressants (TCAs), and monoamine oxidase inhibitors (MAOIs) lowers glutamatergic activity in some regions, such as the hippocampus (61, 64), while the acute administration of NMDA receptor antagonists produces anti-anxiety and antidepressant effects in preclinical and clinical models (43, 62). Lamotrigine, used in epilepsy and bipolar depression, inhibits glutamate release and has proven efficacy in certain symptoms of post-traumatic stress disorder (PTSD), namely, re-experiencing and avoidance/numbing (65). Similarly, topiramate, which acts partly as an AMPA/kainate blocker, decreases re-experiencing symptoms in PTSD (66).

### Glutamate Receptor Dysregulation Contributes to Schizophrenia

Animal models of schizophrenia have implicated glutamate receptor dysregulation and other proteins relating to glutamate transmission, including EAAT2 (54). The glutamate hypothesis of schizophrenia is supported by the finding that the NMDA receptor antagonist phencyclidine blocks glutamate-activated postsynaptic currents and induces schizophrenia-like symptoms, including psychosis and cognitive impairment (50). Moreover, phencyclidine impairs prepulse inhibition (PPI) of the startle reflex, a simple form of information processing that is consistently reduced in schizophrenia. In rats treated with ceftriaxone and acute phencyclidine, PPI was more impaired compared with rats given either treatment alone (67). A broad-spectrum cephalosporin antibiotic, ceftriaxone stimulates EAAT2 expression and lessens neurotoxicity by inhibiting neuronal cell death associated with glutamate excitotoxicity (68). Upregulation of EAAT2 expression by ceftriaxone is thought to be via presynaptic activation of the mGluR, as mGluR2/3 agonist treatment prevented the PPI impairment associated with ceftriaxone-induced upregulation of EAAT2 in rats (69). EAAT2 overexpression causing PPI impairment may be due to glutamate spillover, which mGluR2/3 relies on for activation due to its perisynaptic localization (54). In patients with schizophrenia, oral clozapine 25–35 mg/kg/day for 3 weeks downregulated astrocytic EAAT2 levels and increased extracellular glutamate levels (70).

Interestingly, expression of the presynaptic protein synaptophysin, which is involved in neurotransmitter release, is significantly increased in the same anatomical areas where EAAT2 levels are downregulated by clozapine (71). This suggests that glutamate release may assist clozapine in potentiation of the excitatory synapse. More support for glutamate receptor dysregulation in schizophrenia is seen with the selective GlyT1 inhibitor sarcosine (N-methylglycine), which has shown promise in the treatment of cognitive impairment in patients with chronic schizophrenia (56, 72).

### The Dysregulated Glutamate System and AD

It is well-known that AD is associated with reductions in glutamate transporter capacity and protein expression, as well as a selective loss of the vesicular glutamate transporter (73–75). Recent evidence further points to impairment of EAAT2 function in AD, with findings of significantly lower levels of EAAT2 gene expression in the cortex and decreased EAAT2 immunoreactivity in the motor cortex of patients with AD (76). Although EAAT2b expression did not vary significantly according to disease severity, significantly upregulated levels of exon-skipping variant mRNA expression have been found, which reduces wild-type EAAT2 protein expression in primary astrocytes and inhibits glutamate transport (76). The correlation of EAAT2 expression with increasing neurodegeneration, in combination with the ability of exon-skipping variants to reduce glutamate reuptake, suggests that increased glutamate levels may propagate excitotoxic processes implicated in AD pathogenesis.

Moreover, iGluRs may also contribute to the excitotoxic processes implicated in AD pathogenesis. It is well-established that the NMDA receptor plays an important role in excitotoxicity (77). Hyperactivity of the NMDA receptor may result in a flooding of cations (e.g., Ca^2+^) into the neuron leading to degeneration of the dendritic spines or even the death of the neuron. In patients with AD, treatment with the uncompetitive NMDA receptor antagonist memantine can benefit the cognitive symptoms of AD (78). Notably, in preclinical models of AD, AMPA-mediated transmission, and altered synapse morphology correlated with cognitive decline (47). An initial loss of dendritic spines and synapses is accompanied by a concomitant increase in presynaptic release probability, as the neuronal circuit attempts to compensate for synaptic dysfunction and loss (47). This evidence suggests that increased synaptic glutamate levels induced by a reduction in reuptake may trigger iGluR hyperactivity and lead to the excitotoxic processes relating to the cognitive symptoms of AD.

## Acupuncture Modulates Glutamate Neurotransmission

To date, very little neuroscience research has explored the effects of acupuncture on the role of glutamate in neuropsychiatric disorders. While studies of acupuncture analgesia indicate that acupuncture stimulation may modulate levels of expression of glutamate expression and its receptor, as well as EAAT expression (79, 80), no existing studies have reported on acupuncture-induced modulation of the glycine transporter or other upstream regulatory mechanisms (e.g., D-amino acid oxidase and the amino acid transporter system).

Research has reported that both high- and low-frequency EA significantly decreases upregulated levels of NMDA receptor 1 and 2A and AMPA receptor 1 expression in the spinal cord in an inflammatory pain animal model (81). These findings are corroborated by other research reporting that EA (10 Hz) inhibited phosphorylation of NMDA receptor 1 in spinal cord and alleviated pain in a rat model of inflammatory pain (82). At the supraspinal level, alternating high- and low-frequency EA decreased levels of NMDA receptor 1 and c-fos expression in the rostral ventromedial medulla in an animal model of visceral pain (83). Thus, the expression of glutamate and NMDA receptors may be modulated by acupuncture stimulation in the CNS.

Acupuncture also modulates EAAT2 expression. A recent study examined the effect of acupuncture on depressive behaviors and EAAT2 in rats subjected to chronic unpredictable mild stress (44). Both acupuncture therapy and drug treatment with the glutamate reuptake enhancer riluzole significantly increased sucrose consumption in the sucrose preference test paradigm. This increase in sucrose consumption was associated with an elevated food intake and shortened latency in the novelty-suppressed feeding test paradigm. The amelioration of depressive behavioral actions was consistent with increasing numbers of EAAT2-positive cells and protein expression in the hippocampus and prefrontal cortex. EAAT2 mRNA expression was also increased in the prefrontal cortex, but there was no change in the hippocampus. Moreover, the antidepressant effect was observed later with acupuncture than with riluzole, indicating that repeated acupuncture stimulation may be needed to accumulate EAAT2 expression. Thus, acupuncture-mediated modulation of EAAT2 expression may ameliorate depression.

## Conclusions

In summary, evidence indicates that acupuncture treatment may be of benefit in several neuropsychiatric disorders, including depression, anxiety, schizophrenia, and AD. The pathophysiology of these disorders may be associated with glutamate dysregulation, marked by a high rate of glutamate release and elevated expression of glutamate receptors and glutamate transporters in the CNS. The ability of acupuncture stimulation to modulate glutamate receptor and EAAT expression suggests that the treatment effects of acupuncture are underpinned by its intervention in the dysregulated glutamate system. Further preclinical and clinical studies are needed to clarify the possible mechanisms of acupuncture in these neuropsychiatric disorders and to establish protocols for treatment guidelines.

## Author Contributions

C-HT and IM: collecting and analyzing literature and writing the manuscript; Y-HC: designing and coordinating the study as well as writing the manuscript.

### Conflict of Interest Statement

The authors declare that the research was conducted in the absence of any commercial or financial relationships that could be construed as a potential conflict of interest.
